# Electrical stimulation therapy of the lower esophageal sphincter is successful in treating GERD: long-term 3-year results

**DOI:** 10.1007/s00464-015-4539-5

**Published:** 2015-10-20

**Authors:** Leonardo Rodríguez, Patricia A. Rodriguez, Beatrice Gómez, Manoel Galvao Netto, Michael D. Crowell, Edy Soffer

**Affiliations:** Centro Clinico de Obesidad Diabetes y Reflujo, Santiago, Chile; Department of Surgery, Gastro Obeso Center, Sao Paulo, Brazil; Department of Gastroenterology, Mayo Clinic, Scottsdale, AZ USA; Department of Medicine, Keck School of Medicine at the University of Southern California, Los Angeles, CA USA

**Keywords:** GERD, Treatment, Electrical stimulation, Lower esophageal sphincter, Esophageal acid, Outcomes

## Abstract

**Background:**

Electrical stimulation of the lower esophageal sphincter (LES) has been shown to improve outcomes in patients with gastroesophageal reflux disease (GERD) at 2 years. The aim of the study was to evaluate the safety and efficacy of LES stimulation in the same cohort at 3 years.

**Methods:**

GERD patients with partial response to PPI, with % 24-h esophageal pH < 4.0 for >5 %, with hiatal hernia <3 cm and with esophagitis ≤LA grade C were treated with LES stimulation in an open-label 2-year trial. All patients were on fixed stimulation parameter of 20 Hz, 220 μs, 5 mA delivered in twelve, 30-min sessions. After completing the 2-year open-label study, they were offered enrollment into a multicenter registry trial and were evaluated using GERD-HRQL, symptom diaries and pH testing at their 3-year follow-up.

**Results:**

Fifteen patients completed their 3-year evaluation [mean (SD) age = 56.1 (9.7) years; men = 8] on LES stimulation. At 3 years, there was a significant improvement in their median (IQR) GERD-HRQL on electrical stimulation compared to both their on PPI [9 (6–10) vs. 1 (0–2), *p* = 0.001] and off PPI [22 (21–24) vs. 1 (0–2), *p* < 0.001]. Median 24-h distal esophageal acid exposure was significantly reduced from [10.3 (7.5–11.6) % at baseline vs. 3 (1.9–4.5) %, *p* < 0.001] at 3 years. Seventy-three % (11/15) patients had normalized their distal esophageal acid exposure at 3 years. Remaining four patients had improved their distal esophageal acid exposure by 39–48 % from baseline. All but four patients reported cessation of regular PPI use (>50 % of days with PPI use); three had normal esophageal pH at 3 years. There were no unanticipated device- or stimulation-related adverse events or untoward sensation reported during the 2- to 3-year follow-up.

**Conclusion:**

LES-EST is safe and effective for treating patients with GERD over long-term, 3-year duration. There was a significant and sustained improvement in esophageal acid exposure and reduction in GERD symptoms and PPI use. Further, no new GI side effects or adverse events were reported.

Gastroesophageal reflux disease (GERD) is a chronic disease with high global prevalence of up to 20 % [1]. GERD occurs when a weak or dysfunctional lower esophageal sphincter (LES) exposes the esophagus to acidic stomach contents, resulting in bothersome symptoms of heartburn, regurgitation, and chest pain and a significant impairment of patients’ quality of life [2]. Acid damage can lead to stricture formation and Barrett esophagus, which can eventually lead to esophageal adenocarcinoma, one of the fastest rising cancers in the Western world [3]. Excessive esophageal acid exposure is the hallmark of this disease, and measurement of 24- to 48-h esophageal acid exposure is the most robust gold-standard for the diagnosis of GERD [4].

Current therapies aim to treat GERD by controlling or eliminating esophageal acid exposure. Medications such as proton pump inhibitors (PPI) minimize esophageal acid exposure by blocking gastric acid secretion; however, due to persistent LES dysfunction, reflux of non-acidic gastric contents continues at comparable magnitude, resulting in persistent symptoms in up to 40 % of patients [5]. Laparoscopic fundoplication for sphincter augmentation controls reflux of all gastric contents; however, the anatomical alteration at the level of the GE junction is associated with multiple side effects, some debilitating, that plague the procedure [6], as well as recurrence of symptoms in a sizable number of patients [6, 7]. Concerns about optimal outcomes in low-volume centers persist [8, 9]. Perhaps as a result, the number of fundoplication procedures performed has been declining [10].

An ideal antireflux procedure should result in long-term control of esophageal acid exposure, without any significant side effects or adverse events, thus improving patients’ symptoms and quality of life without dependence on daily medications [11]. We had previously reported the results from our long-term, 2-year open-label trial of LES stimulation in patient of GERD [12]. The current report describes the results of stimulation therapy after extension of the follow-up in the same cohort of patients to 3 years.

## Materials and methods

**Fig. 1:**
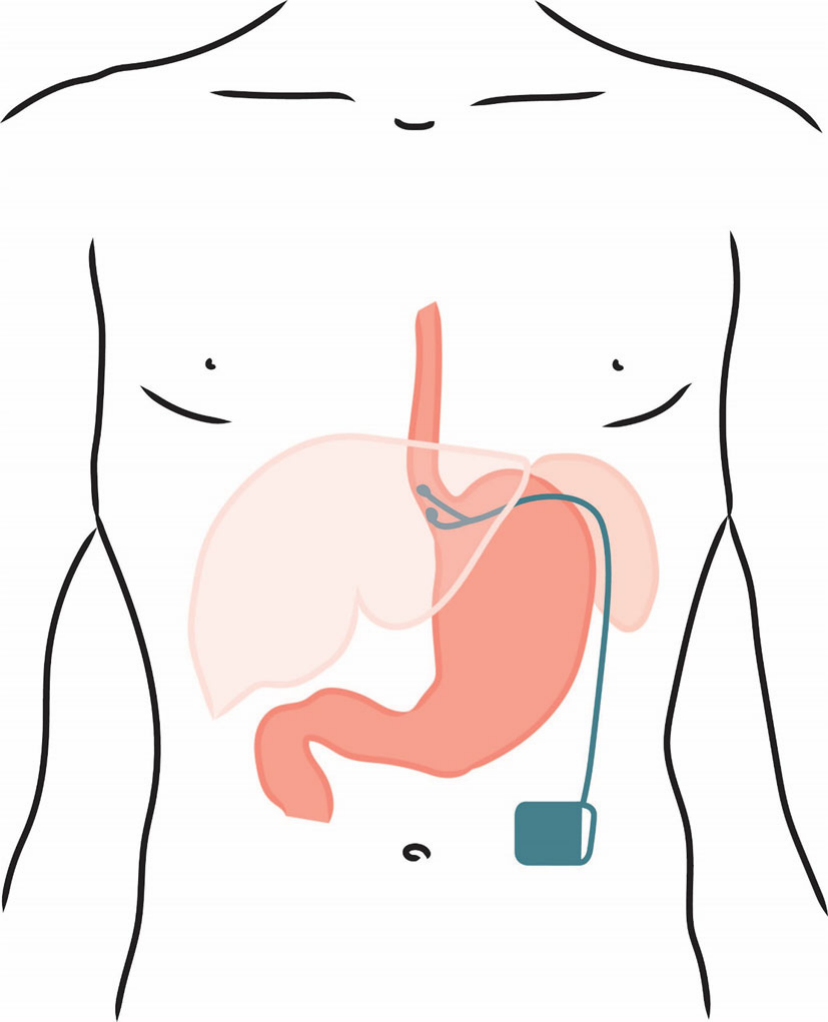
LES stimulation system with electrodes implanted in the LES and the pulse generator implanted in a subcutaneous pocket in the anterior abdominal wall

A Web-based international multicenter registry has been established by EndoStim (The Hague, The Netherlands), the manufacturer of the LES stimulation system, to allow physicians to track the outcomes of their patients treated with LES stimulation in their clinical practice outside of clinical trials (NCT02441400). GERD patients with partial response to PPI, with hiatal hernia ≤3 cm and with ≤grade C esophagitis were enrolled in a single-center, open-label trial of electrical stimulation of the LES using the EndoStim^®^ LES stimulation system (Fig. 1). The system is implanted laparoscopically and is programmed to deliver intermittent stimulation to the LES. The details of the open-label trial and the 2-year results have been previously reported [12]. At the end of the 2-year follow-up, patients were offered to participate in a registry trial to allow for a follow-up of up to 5 years, to provide an assessment of long-term clinical outcomes. The registry trial was approved by the Institutional Review Board of Clinica Indisa, Santiago, Chile. At their 3-year follow-up, patients who enrolled in the registry trial were offered evaluation using GERD-HRQL, and daily diary questionnaires, SF-12, as well as esophageal pH testing to objectively measure their esophageal acid exposure.

The effect of electrical stimulation of the LES on patient symptoms was measured using GERD-HRQL (on PPI and 2 weeks off PPI at baseline and after 2 weeks off PPI during follow-up), symptoms and medication use reported in a daily diary, and general quality of life measured using SF-12 (on PPI and 2 weeks off PPI at baseline and after 2 weeks off PPI during follow-up visits) using related samples Wilcoxon signed-rank test was used for comparison. Change in % time distal esophageal pH was <4.0 was assessed by comparing the results at baseline to the 3-year follow-up. LES electrical stimulation therapy (EST) was evaluated using related samples Wilcoxon signed-rank test. A *p* value of <0.05 was considered statistically significant.

## Results

**Fig. 2:**
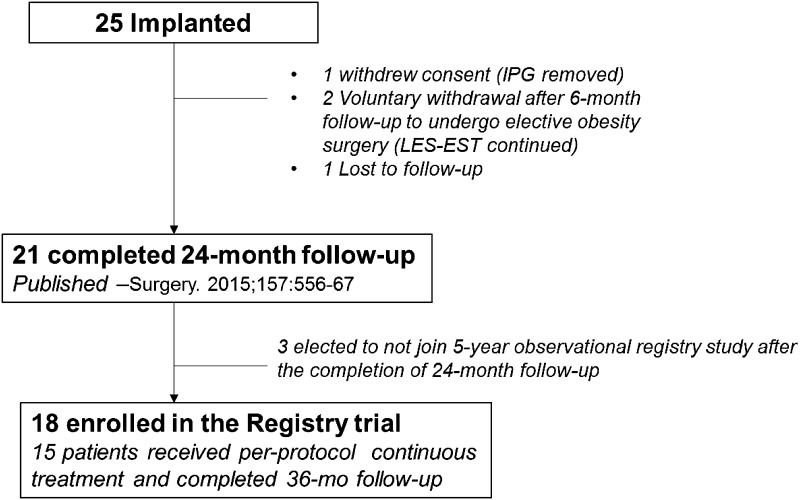
Patient follow-up chart

Eighteen patients consented to enroll in the registry trial, and 15 of those patients were available for evaluation at 3 years. The details of patient follow-up are presented in Fig. 2, and the baseline characteristics of patients that underwent 3-year evaluation are provided in Table 1.

**Table 1:** Baseline characteristics and relevant medical/GERD history of patient treated per-protocol with LES stimulation and completing their 3-year evaluation

Characteristic	*N*	Mean (SD)
Age (years)	15	56.1 (9.7)
Body mass index (BMI)	15	27.4 (3.2)
*Gender*		
Male	8	
Female	7	
*BMI class*		
Normal (<25)	3	
Overweight (≥25 and <30)	9	
Obese (≥30)	3	

Patients using daily PPI	100 %	
Duration of GERD symptoms		
Mean (SD)	>12.2 (9.1) years	
Median (quartile)	>10 (7.5–12.5) years	
Duration of PPI use		
Mean (SD)	>5.9 (3.3) years	
GERD-HRQL Total Score		
On PPI		
Median (IQR)	9 (6–10)	
% not satisfied	80 %	
Off PPI		
Median (IQR)	22 (20.5–24)	
% not satisfied	94 %	
Total % pH time <4, Median (IQR)	10.3 (7.5–11.6)	
DeMeester Score, Median (IQR)	36.9 (30.8–44.3)	
Esophagitis (%) grade A/B/C	60/33.3/6.7	
Hiatal hernia (%) none/<2 cm/≥2 cm	93.3/0/6.7	

*SD* = standard deviation, *IQR* interquartile range

There were no additional adverse events reported between years 2 and 3 of follow-up. The details of adverse events prior to 2 years have been previously reported [13] and include two serious adverse events: acute non-cardiac retrosternal chest pain and surgery for solitary thyroid nodule, both deemed not related to device or procedure by an independent data safety and monitoring board. There were a total of 63 non-serious adverse events in the trial; 12 were classified as device/procedure related. Most device-/procedure-related events were typical of a surgical implant procedure (e.g., implant site pain, post-op nausea), mild–moderate in severity and resolved with or without intervention. There were no stimulation-related side effects or sensation, and no events of dysphagia were reported.

**Fig. 3:**
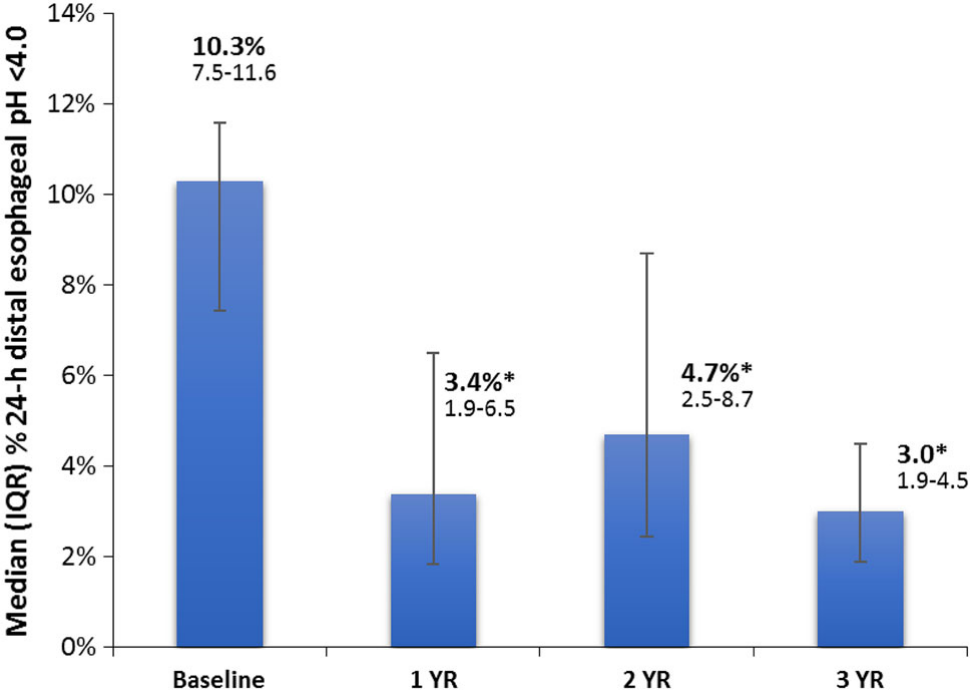
Sustained improvement in the distal esophageal acid exposure on LES stimulation at 3-year follow-up. Data: median, IQR. 73 % reported normalization (<4.0 % of 24 h) in their distal esophageal acid exposure at their 3-year follow-up

The median distal esophageal acid exposure was significantly improved at 3 years at 3 % (IQR 1.9–4.5) compared to 10.1 % (7.8–13; *p* < 0.001) in the whole cohort and 10.3 % (4.0–7.8; *p* < 0.001) in the matched cohort at baseline (Fig. 3). Seventy-three % (11/15) reported normalization (<4.0 % of 24 h) of their distal esophageal acid exposure. The remaining four patients had 39–48 % improvement in their distal esophageal acid exposures. The median DeMeester score was significantly improved at 3 years from 36.9 (30.8–44.3) at baseline to 12.8 (7.2–18.8; *p* = 0.0003).

**Fig. 4:**
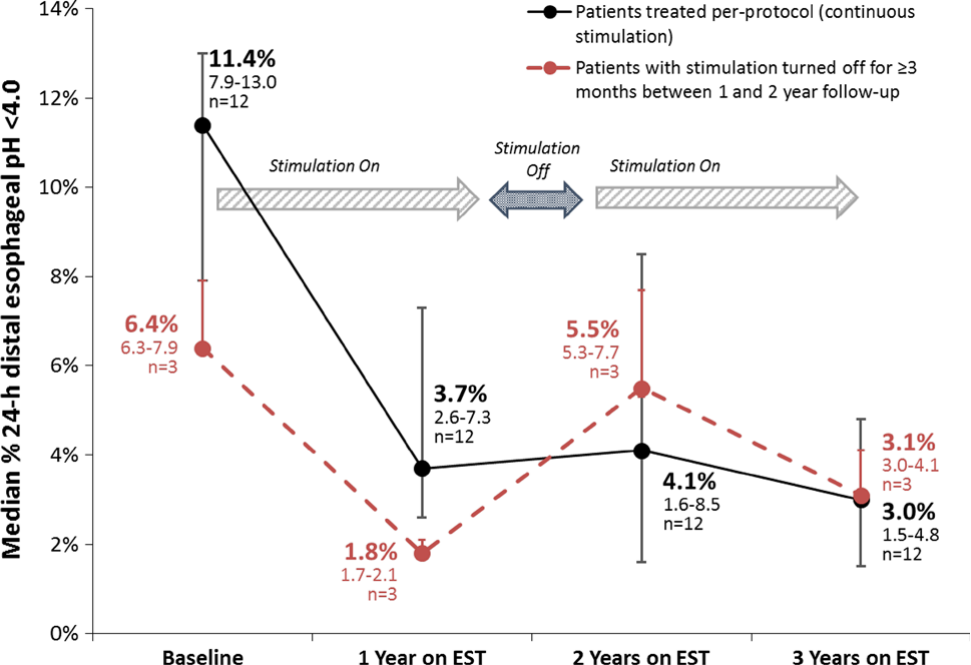
Effect of blinded turn-off and turn-on on esophageal acid exposure. Esophageal acid exposure increased on blinded turn-off before the 2-year pH study and then improved after blinded turn-on at 2 years, as measured at their 3-year follow-up. *EST* electrical stimulation therapy

Three patients underwent blinded turn-off of LES-EST after their 18-month follow-up, and one patient had her therapy accidentally turned-off by inadvertent use of a magnet therapy for her arthritis at month 15. All four patients demonstrated worsening of their distal esophageal acid exposure at 2 years compared to their on-therapy at 12 months, though values improved, they did not return to their baseline esophageal acid exposure even after >3 months of cessation of LES-EST [12]. All these patients had their stimulation turned back after their 2-year visit, and three of these patients underwent esophageal pH testing at 3 years. All three patients had significantly improved or normalized their distal esophageal acid exposure, suggesting a causal association between improvement in esophageal acid exposure and LES stimulation (Fig. 4).

**Fig. 5:**
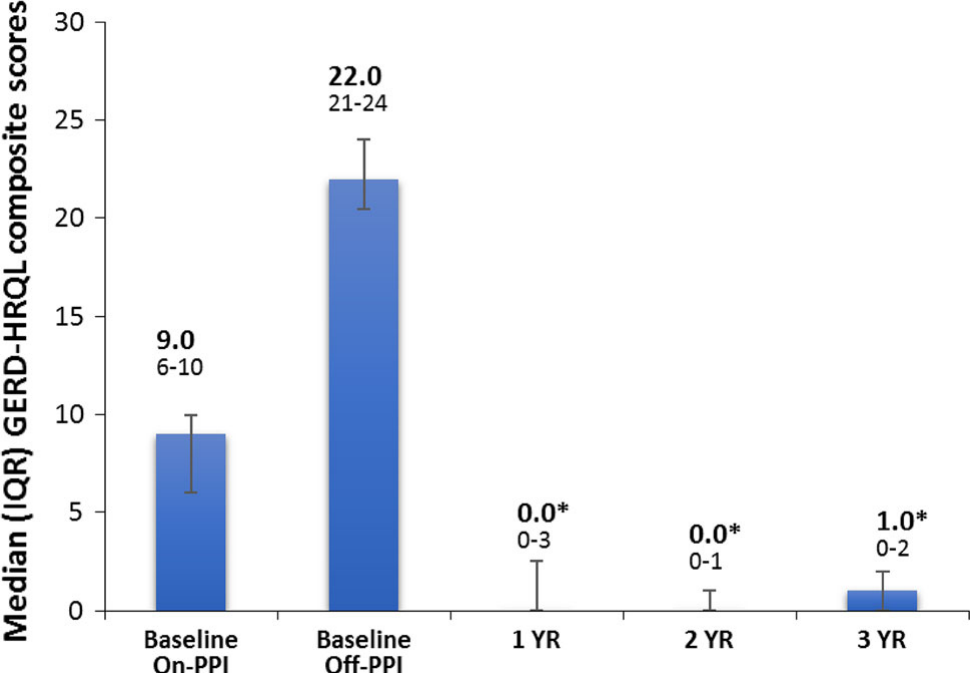
Sustained improvement in GERD symptoms as measured by the composite GERD-HRQL scores at 3-year follow-up. Data: median, IQR. All patients reported clinically significant improvement (≥50 % improvement in the composite GERD-HRQL score) in symptoms at 3 years compared to baseline off PPI and better composite GERD-HRQL scores than baseline on PPI

**Fig. 6:**
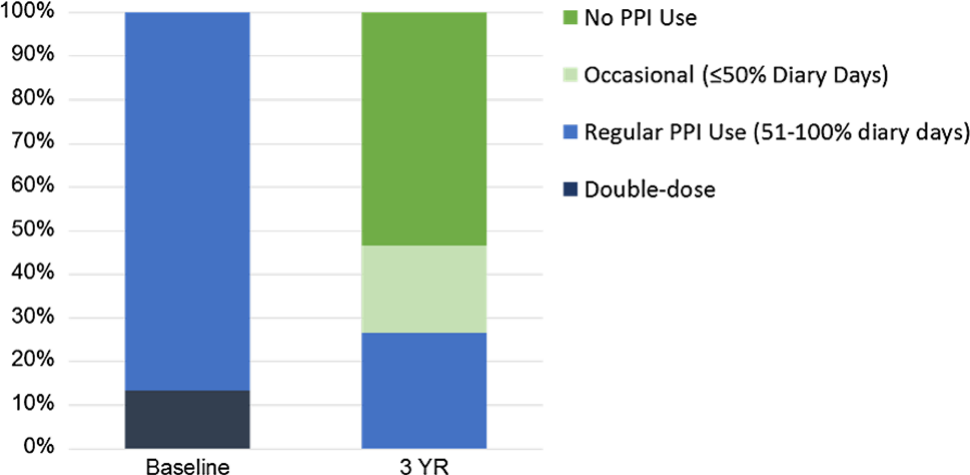
PPI medication use at baseline and at 3-year follow-up. Most patients (73 %) were free from PPI dependence (dependence defined as ≥50 % diary days with PPI use)

The median composite GERD-HRQL scores were significantly improved at 3 years at 1.0 (IQR 0–2.0) compared to 23.5 (21–25; *p* < 0.001) off PPI and 9.0 (6–10; *p* < 0.001) on PPI, respectively, in the whole cohort and 22 (21–24; *p* < 0.001) off PPI and 9.0 (6–10; *p* < 0.001) on PPI, respectively, in the matched cohort at baseline (Fig. 5). All patients reported clinical significance (> 50 % improvement) in their composite GERD-HRQL score versus both their baseline off-PPI scores and on-PPI scores. The SF-12 scores for both physical and mental health improved numerically compared with baseline on- and off-PPI scores, but did not reach statistical significance. There was a significant reduction in dependence on PPI medications. All patients at baseline were on either single- or double-dose PPI for a median duration of 6.0 years. Seventy-three % of patients were free of PPI dependence at their 3-year follow-up (Fig. 6). Three patients were using daily PPI, two had normal distal esophageal acid exposure, and one was improved by 39 %, while one patients was using PPI 4X/week and had normal distal esophageal acid exposure. All esophageal pH measurements were off PPI.

All patients in the cohort had erosive esophagitis at baseline. Twelve patients underwent endoscopy at their 3-year follow-up with 50 % (6/12) patients showing improvement in their esophagitis by ≥1 grade, and 25 % (3/12) patients each had stable grade A and worsening (grade A to grade B) esophagitis. Two of the 3 patients that had worsening esophagitis and all 3 patients with stable esophagitis had normal esophageal acid exposure at their 3-year evaluation. None of the patients had developed Barrett esophagus at their 3-year follow-up on LES stimulation therapy.

## Discussion

This is the first report of sustained control of esophageal acid exposure at 3 years with LES electrical stimulation in patients with GERD, who were at least partially responsive to PPI at baseline evaluation. Along with objective improvement in GERD, there was sustained improvement in GERD symptoms and GERD-related quality of life, and elimination or reduction in need for daily PPI medications, without any new adverse events.

Excessive esophageal acid exposure due to the failure of the antireflux barrier is the hallmark of GERD. The current gold-standard objective test to diagnose GERD is the prolonged ambulatory esophageal pH-metry [14]. The total time with esophageal pH < 4 as recorded by a probe placed 5 cm above the LES and the composite Johnson-DeMeester score have the highest sensitivity and specificity in the diagnosis of GERD [4]. Control of esophageal acid exposure is the most robust measure of effectiveness of a GERD therapy, although pH testing has not been an endpoint for most GERD therapy clinical trials. Our results show a significant and sustained improvement in esophageal acid exposure and Johnson-DeMeester composite scores with LES stimulation at 3-year follow-up. Almost 3/4th of the patients, mostly partial responders, evaluated at 3-year follow-up on LES stimulation therapy had normalized their esophageal acid exposure, while the remaining 1/4th had improved (39–48 %) but not normalized esophageal acid exposure. This important observation highlights the durability of acid exposure control with LES stimulation over time. Sustained long-term control of esophageal acid exposure is essential for any effective GERD therapy. This contrasts with the abnormal esophageal pH values documented in 56 % of patients with refractory heartburn on twice daily PPI, and in 30 % of patients with typical GERD symptoms who were well controlled on once daily PPI [15, 16]. In another study, 50 % of patients with GERD, who achieved complete symptom control with PPI therapy, were found to have abnormal esophageal exposure while on PPI [17].

In addition to the objective improvement in esophageal acid exposure, there was also a significant and sustained improvement in patient-centric outcomes of GERD symptoms, both on and off PPI as well as acid suppression medication use at 3 years on LES stimulation. Almost two-thirds of the patients chose to undergo antireflux surgery due to incomplete symptom control with medication, and the rest were unwilling to take medications due to quality of life, safety or cost concerns. The proportion of patients and physicians concerned about long-term safety of high-dose PPI, and the literature on this topic is growing, resulting in an increasing number of patients and physicians who desire an alternative to long-term PPI [18]. Dependence on daily PPI was eliminated in 80 % with additional 20 % using PPI, albeit at a reduced dose. Although elimination of any PPI use in all patients is the most desirable outcome, a significant reduction in the cumulative dose is also quite beneficial, as some observational studies showed that the adverse effect profile of PPI is dose dependent [19].

Finally, the greatest advantage of electrical stimulation is its safety profile, observed in other applications over the years. Except for anticipated events typically seen after a laparoscopic implant procedure, no other significant adverse events have been reported in our patients. More importantly, due to the minimal anatomical disruption with the electrode implant procedure, the usual side effects of dysphagia, gas bloat or diarrhea seen with traditional antireflux surgery were not encountered in our patients [6]. An ongoing international multicenter trial has reported comparable results in a more diverse patient population and across multiple operators [20]. Thus far in our experience, the safety profile of LES stimulation appears to be quite superior to traditional antireflux surgery [6]. However, continuous monitoring of any safety events in a larger group of patients, treated across multiple practice settings over a longer follow-up period, is required to conclusively establish the safety of this therapy.

There are limitation of our study, primarily the small number of patients and open-label study design. We believe that the profound improvement in 24-h esophageal acid exposure sustained over a 3-year period is highly unlikely to be a placebo response [21]. Additionally, our blinded turn-off and turn-on sub-study points to a causal effect of LES stimulation on improvement in the esophageal acid exposure. A larger experience in diverse group of patients and operators is needed to validate our trial results for a wider application of this procedure. Such data are currently being collected in the international multicenter trial and in routine clinical practice in an international multicenter registry. A well-designed sham control trial would be helpful in overcoming the limitations of an open-label design.

Comparative effectiveness study against standard antireflux surgery has been recently discussed [22, 23]. However, such a study is likely to be quite difficult, given issues of patient preference and generalizability of the results to less stringent patient population and operators. We believe that the LES stimulation therapy fills a “therapy gap” or an “under-met need” between pharmacotherapy and traditional antireflux surgery, particularly given its excellent safety profile. Additionally, due to the lack of any negative effect on esophageal body or LES motor function demonstrated on high-resolution manometry, LES stimulation therapy may be a desirable option in specific patient populations such as those with severe esophageal dysmotility including aperistalsis, post-myotomy GERD in patients with achalasia and in patients with GERD following laparoscopic sleeve gastrectomy for obesity. Early experience suggests that LES stimulation maybe effective in the latter group of patients.

In conclusion, at 3-year follow-up LES stimulation therapy effectively controls esophageal acid exposure and eliminates GERD symptoms and the need for regular PPI medications in majority of GERD patients that were at least partial PPI responder. LES stimulation had no long-term side effects and was associated with minimal adverse events mainly restricted to the post-op period. We believe that based on our long-term results and those being reported from the international multicenter trial [20], LES stimulation could be a therapeutic option for well-informed and select patients with GERD who are seeking an alternative to current medical or surgical therapies for GERD.
